# Crystal structure and Hirshfeld surface analysis of (*E*)-1-(3,5-di­chloro-2-hy­droxy­phen­yl)-3-(5-methyl­furan-2-yl)prop-2-en-1-one

**DOI:** 10.1107/S2056989018012173

**Published:** 2018-09-18

**Authors:** N. R. Sreenatha, B. N. Lakshminarayana, D. P. Ganesha, C. R. Gnanendra

**Affiliations:** a Department of Physics, Adichunchanagiri Institute of Technology, Chikamagaluru 577 102, Karnataka, India; b Department of Physics, Government Engineering College, Hassan 573 201, Karnataka, India; c Department of Chemistry, Adichunchanagiri Institute of Technology, Chikamagaluru 577 102, Karnataka, India

**Keywords:** crystal structure, chalcones, furan, hydrogen bonding, Hirshfeld surfaces, fingerprint plots

## Abstract

The title chalcone derivative, C_14_H_10_Cl_2_O_3_, is almost planar, with a dihedral angle of 7.0 (2) ° between the 3,5-di­chloro-2-hy­droxy­phenyl and 5-methyl­furan rings. There is an intra­molecular O—H⋯O hydrogen bond present forming an *S*(6) ring motif. In the crystal, mol­ecules are linked by bifurcated C—H/H⋯O hydrogen bonds, enclosing an *R*_1_^2^(6) ring motif, forming a 2_1_ helix propagating along the *b*-axis direction. The inter­molecular inter­actions were qu­anti­fied using Hirshfeld surface analysis.

## Abstract 2

The title chalcone derivative is almost planar, with a dihedral angle of 7.0 (2)° between the 3,5-di­chloro-2-hy­droxy­phenyl and 5-methyl­furan rings.

## Chemical context   

Chalcone derivatives are an important class of organic compounds comprising two aromatic rings connected *via* an α,β unsaturated carbonyl system. They belong to the flavonoid family, which are basically found in fruits and vegetables (Hijova 2006 ▸). Chalcones occupy an important place in the pharmaceutical industry since their derivatives serve as the core structures for many organic compounds possessing various biological activities such as anti­bacterial (Vibhute & Baseer, 2003 ▸), anti-microbial (Prasad *et al.*, 2006 ▸), anti-inflammatory (Lee *et al.*, 2006 ▸), anti-hyperglycemic (Satyanarayana *et al.*, 2004 ▸), anti-malarial (Syahri *et al.*, 2017 ▸) and anti-oxidant (Cheng *et al.*, 2008 ▸). Chalcones also exhibit some non-linear optical (NLO) properties and also find applications in laser technologies such as optical communications, data storage and signal processing because of the α,β unsaturated functionality (Shobha *et al.*, 2017 ▸). Based on the above importance, we report here the crystal structure of (*E*)-1-(3,5-di­chloro-2-hy­droxy­phen­yl)-3-(5-methyl­furan-2-yl)prop-2-en-1-one.
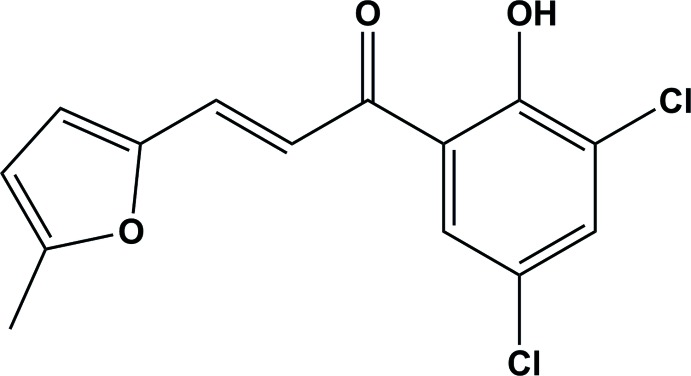


## Structural commentary   

**Figure 1:**
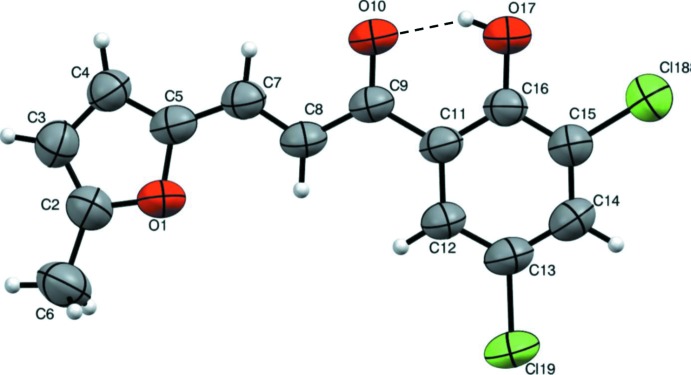
The mol­ecular structure of the title compound, with atom labelling and 50% probability displacement ellipsoids. The intra­molecular hydrogen bond (Table 1 ▸) is indicated by a dashed line.

The title mol­ecule comprises 5-methyl­furan and 3,5-di­chloro-2-hy­droxy­phenyl rings connected *via* an unsaturated α,β carbonyl system as shown in Fig. 1 ▸. The mol­ecule is relatively planar with the furan and benzene rings being inclined to each other by 7.0 (2)°. There is an intra­molecular O—H⋯O hydrogen bond present forming an *S*(6) ring motif (Table 1 ▸ and Fig. 1 ▸). The chlorine atoms positioned at C13 and C15 of the phenyl ring are in an -*anti*-periplanar conformation described by the torsion angles C11—C12—C13—Cl19 = −179.1 (3)° and C13—C14—C15—Cl18 = −178.6 (4)°, while methyl group at C2 of the furan ring is in a +anti-periplanar conformation [C5—O1—C2—C6 = 178.3 (4)°]. The bond lengths and angles in the title compound are similar to those observed for 3-(furan-2-yl)-1-(2-hy­droxy­phen­yl)prop-2-en-1-one (Kong & Liu, 2008 ▸).

## Supra­molecular features   

**Figure 2:**
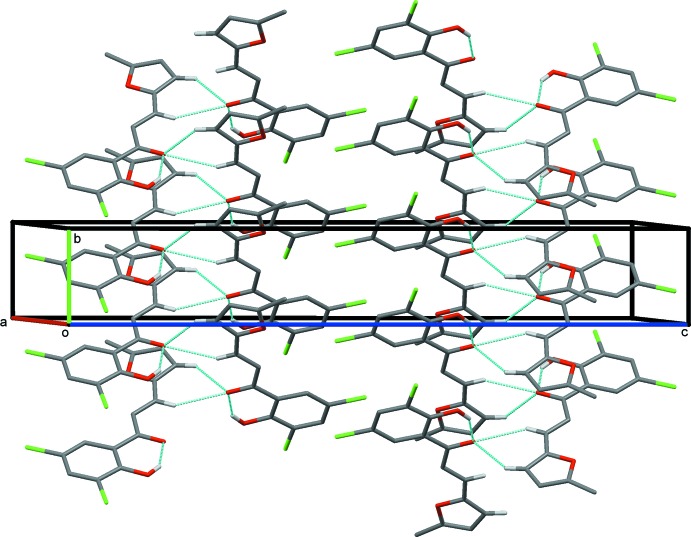
A view normal to the *bc* plane of the crystal packing of the title compound. The hydrogen bonds (Table 1 ▸) are shown as dashed lines and only the H atoms involved in these inter­actions are shown.

**Figure 3:**
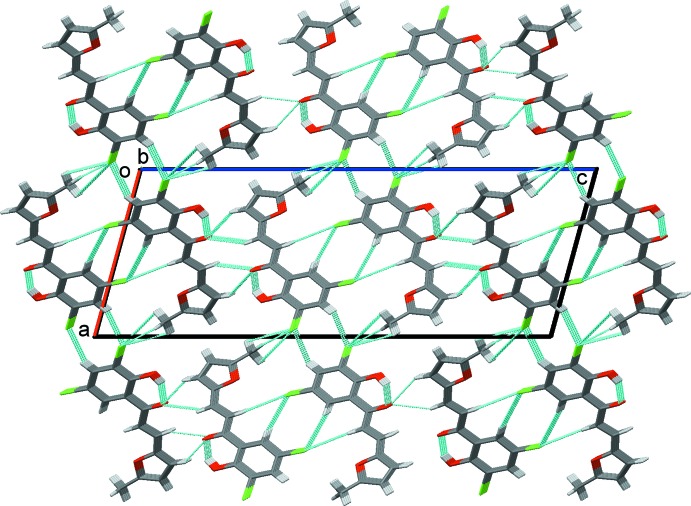
A view along the *b* axis of the crystal packing of the title compound. The hydrogen bonds (Table 1 ▸) and short contacts (Table 2 ▸) in the crystal structure are shown as dashed lines.

In the crystal, mol­ecules are linked by bifurcated C—H/H⋯O hydrogen bonds, enclosing an 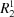
(6) ring motif, forming a 2_1_ helix with a pitch of 4.402 (1) Å, propagating along the *b*-axis direction (Table 1 ▸, Fig. 2 ▸). The helices appear to be linked by very weak inter­molecular C—H⋯Cl contacts (Table 2 ▸ and Fig. 3 ▸; see also Fig. 6 ▸ and the section below).

## Hirshfeld surfaces and 2D fingerprint analysis   

Three-dimensional Hirshfeld surfaces and their associated two-dimensional fingerprint plots are used to analyze inter­molecular inter­actions in crystal structures. The Hirshfeld surfaces are unique for every crystal structure based on spherical atomic electron densities and are obtained using the *CrystalExplorer* software (Spackman & Jayatilaka 2009 ▸).

**Figure 4:**
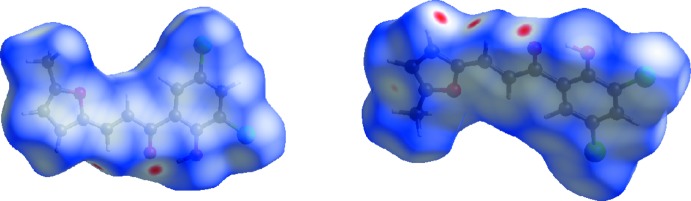
The Hirshfeld surface mapped over *d*_norm_ in the range −0.1183 to +1.0844 a.u. The circular red spots indicate inter­molecular C—H⋯O inter­actions.

**Figure 5:**
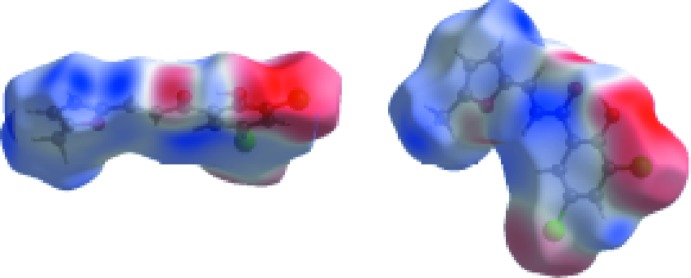
The Hirshfeld surface mapped over electrostatic potential in the range −0.0506 to +0.0422 a.u. The donor and acceptor atoms participating in these inter­actions are shown respectively as positive (blue regions) and negative electrostatic potentials (red regions).

The three-dimensional Hirshfeld surface was mapped over *d*_norm_ using a red–blue–white colour scheme where the red and blue regions indicate contact distances less then and greater than, respectively, the sums of the van der Waals radii, which have negative and positive *d*_norm_ values, respectively. In white regions where *d*_norm_ is zero the contacts are almost equal to the sum of the van der Waals radii (Shaik *et al.* 2017 ▸). The presence of an inter­molecular C—H⋯O inter­action is indicated by a deep-red circular spot on the *d*_norm_ surface (Fig. 4 ▸). In addition, inter­molecular C—H⋯O inter­actions can also be viewed on the Hirshfeld surface mapped over electrostatic potential using a STO-3G basis set at the HF (Hartree–Fock) level of theory (Spackman & McKinnon 2002 ▸; McKinnon *et al.* 2004 ▸) as shown in Fig. 5 ▸. The donor and acceptor atoms participating in these inter­actions are shown respectively as positive (blue regions) and negative electrostatic potentials (red regions).

**Figure 6:**
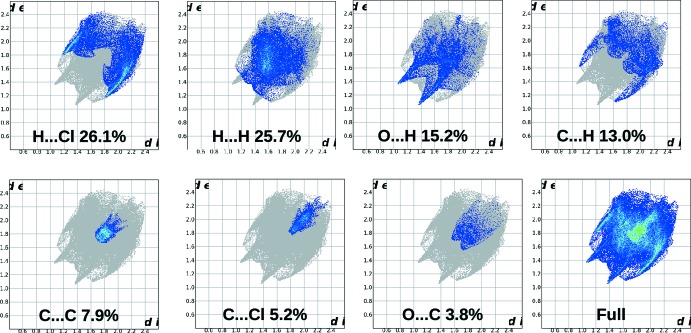
Two-dimensional fingerprints plots.

The two-dimensional fingerprint (Fig. 6 ▸) plots were generated in the expanded mode for all major inter­molecular inter­actions giving their percentage of contribution towards packing of total Hirshfeld surface area for the mol­ecule. The H⋯Cl inter­actions make the highest (26.1%) contribution to the total Hirshfeld surface and appear as a pair of wings in the region 1.2 Å < (*d*_e_ + *d*_i_) < 1.8 Å (*d*_i_ is the distance of a point on the Hirshfeld surface to the nearest nucleus inside the surface while *d*_e_ is the distance of the nearest nucleus outside the surface). The H⋯H contacts, with a contribution of 25.7%, are shown as blue dots spread in the middle region 1.18 Å < (*d*_e_ + *d*_i_) < 1.62 Å. The two sharp spikes observed at 1.04 Å < (*d*_e_ + *d*_i_) < 1.39 Å are due to the presence of a pair of O⋯H contacts making a 15.2% contribution. A pair of C⋯H contacts are observed as characteristic wings in the region of 1.18 Å < (*d*_e_ + *d*_i_) < 1.6 Å (13.0% contribution). C⋯C, C⋯Cl and O⋯C contacts make contributions of 7.9%, 5.2% and 3.8%, respectively.

## Database survey   

A search of the Cambridge Structural Database (CSD, Version 5.39, last update August 2018; Groom *et al.*, 2016 ▸) for 3-(furan-2-yl)-1-(2-hy­droxy­phen­yl)prop-2-en-1-ones gave six hits. These involve only four compounds, namely: 3-(furan-2-yl)-1-(2-hy­droxy­phen­yl)prop-2-en-1-one itself (BOGVID; Kong & Liu, 2008 ▸); 1-(5-bromo-2-hy­droxy­phen­yl)-3-(2-fur­yl)prop-2-en-1-one, for which variable pressure measurements were carried out (KUDMON, KUDMON01, KUDMON02; Bakowicz *et al.*, 2015 ▸); 1,1′-(4,6-dihy­droxy-1,3-phenyl­ene)bis­[3-(2-fur­yl)prop-2-en-1-one] (POHZUJ; Wera *et al.*, 2014 ▸); and 1-(5-acetyl-2,4-di­hydroxy­phen­yl)-3-(2-fur­yl)prop-2-en-1-one (POJBAT; Wera *et al.*, 2014 ▸). As in the title compound there are intra­molecular O—H⋯O hydrogen bonds present forming *S*(6) ring motifs. The mol­ecules are all relatively planar with the dihedral angle between the furan and 2-hy­droxy­phenyl rings varying from *ca* 8.35° in BOGVID, 0.20° in KUDMON, and 10.90 and 2.56° in the two independent mol­ecules of POJBAT. The only exception is POHZUJ, which possesses twofold rotation symmetry and has two [3-(2-fur­yl)prop-2-en-1-one] units *meta* to each other; here the dihedral angle is *ca* 19.87°.

## Synthesis and crystallization   

1-(3,5-Di­chloro-2-hy­droxy­phen­yl)-2-hy­droxy­ethanone (5 mmol) was dissolved in methanol (15 ml) and was stirred with 5 ml of sodium hydroxide solution for 30 min at room temperature. To this mixture, 5-methyl­furan-2-carbaldehyde (5 mmol) was added over 30 min with stirring. Stirring at room temperature was then continued for 32 h. On completion of the reaction, monitored by TLC, the mixture was quenched in ice–water and acidified with dilute hydro­chloric acid. The separated precipitate of the title compound was filtered off and recrystallized from methanol solution giving colourless block-like crystals.

## Refinement   

Crystal data, data collection and structure refinement details are summarized in Table 3 ▸. Hydrogen atoms were placed in calculated positions and refined as riding: C—H = 0.93 Å with *U*_iso_(H) = 1.2*U*_eq_(C) for aromatic H atoms and C—H = 0.96 Å with *U*_iso_(H) = 1.5*U*_eq_(C) for methyl H atoms.

## Supplementary Material

Crystal structure: contains datablock(s) I. DOI: 10.1107/S2056989018012173/qm2127sup1.cif

Structure factors: contains datablock(s) I. DOI: 10.1107/S2056989018012173/qm2127Isup2.hkl

Supporting information file. DOI: 10.1107/S2056989018012173/qm2127Isup3.cml

CCDC reference: 1852049

Additional supporting information:  crystallographic information; 3D view; checkCIF report

## Figures and Tables

**Table 1:** Hydrogen-bond geometry (Å, °)

*D*—H⋯*A*	*D*—H	H⋯*A*	*D*⋯*A*	*D*—H⋯*A*
O17—H17⋯O10	0.82	1.76	2.489 (4)	147
C4—H4⋯O10^i^	0.93	2.54	3.272 (6)	135
C7—H7⋯O10^i^	0.93	2.57	3.359 (4)	143

Symmetry code: (i) 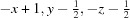.

**Table 2:** Short contacts (Å) in the crystal structure of the title compound *l* − vdW is the length minus the van der Waals separation.

Contact	length	*l* − vdW
O10⋯H17	1.76	−0.96
H4⋯O10^i^	2.54	−0.17
H7⋯O10^i^	2.57	−0.15
H6*A*⋯Cl18^ii^	3.21	+0.26
H6*C*⋯Cl18^ii^	3.21	+0.26
H6*B*⋯Cl18^iii^	3.14	+0.19
Cl19⋯H6*C* ^iv^	3.28	+0.33
Cl19⋯H8^v^	3.13	+0.18
Cl19⋯H12^v^	3.20	+0.25
Cl18⋯H14^vii^	3.28	+0.33

Symmetry codes: (i) 1 − *x*, −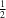 + *y*, −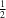 − *z*; (ii) −1 + *x*, −2 + *y*, *z*; (iii) −1 + *x*, −1 + *y*, *z*; (iv) 1 − *x*, −1 − *y*, −*z*; (v) 1 − *x*, −*y*, −*z*; (vi) 2 − *x*, 1 − *y*, −*z*.

**Table 3:** Experimental details

Crystal data	
Chemical formula	C_14_H_10_Cl_2_O_3_
*M*_r_	297.12
Crystal system, space group	Monoclinic, *P*2_1_/*c*
Temperature (K)	290
*a*, *b*, *c* (Å)	10.831 (2), 4.4020 (5), 28.457 (5)
β (°)	105.254 (6)
*V* (Å^3^)	1309.0 (4)
*Z*	4
Radiation type	Mo *K*α
μ (mm^−1^)	0.50
Crystal size (mm)	0.30 × 0.28 × 0.25

Data collection	
Diffractometer	Bruker APEXII
Absorption correction	Multi-scan (*SADABS*; Bruker, 2006 ▸)
*T*_min_, *T*_max_	0.862, 0.906
No. of measured, independent and observed [*I* > 2σ(*I*)] reflections	2940, 2298, 2232
*R*_int_	0.032
(sin θ/λ)_max_ (Å^−1^)	0.595

Refinement	
*R*[*F* ^2^ > 2σ(*F* ^2^)], *wR*(*F* ^2^), *S*	0.061, 0.215, 1.09
No. of reflections	2298
No. of parameters	174
H-atom treatment	H-atom parameters constrained
Δρ_max_, Δρ_min_ (e Å^−3^)	0.30, −0.25

Computer programs: *APEX2* (Bruker, 2006 ▸), *SAINT* (Bruker, 2006 ▸), *SHELXS97* (Sheldrick, 2008 ▸), *SHELXL97* (Sheldrick, 2008 ▸), *PLATON* (Spek, 2009 ▸), *Mercury* (Macrae *et al.*, 2006 ▸).

## supplementary crystallographic information

### Crystal data

**Table:**

C_14_H_10_Cl_2_O_3_	*F*(000) = 608
*M**_r_* = 297.12	*D*_x_ = 1.508 Mg m^−^^3^
Monoclinic, *P*2_1_/*c*	Mo *K*α radiation, λ = 0.71073 Å
*a* = 10.831 (2) Å	Cell parameters from 3210 reflections
*b* = 4.4020 (5) Å	θ = 2.7–25.0°
*c* = 28.457 (5) Å	µ = 0.50 mm^−^^1^
β = 105.254 (6)°	*T* = 290 K
*V* = 1309.0 (4) Å^3^	Block, colourless
*Z* = 4	0.30 × 0.28 × 0.25 mm

### Data collection

**Table:**

Bruker APEXII diffractometer	2298 independent reflections
Radiation source: graphite	2232 reflections with *I* > 2σ(*I*)
Detector resolution: 0.820 pixels mm^-1^	*R*_int_ = 0.032
*SAINT* (Bruker, 2006) scans	θ_max_ = 25.0°, θ_min_ = 2.7°
Absorption correction: multi-scan (SADABS; Bruker, 2006)	*h* = −12→12
*T*_min_ = 0.862, *T*_max_ = 0.906	*k* = −5→4
2940 measured reflections	*l* = −33→33

### Refinement

**Table:**

Refinement on *F*^2^	0 restraints
Least-squares matrix: full	Hydrogen site location: inferred from neighbouring sites
*R*[*F*^2^ > 2σ(*F*^2^)] = 0.061	H-atom parameters constrained
*wR*(*F*^2^) = 0.215	*w* = 1/[σ^2^(*F*_o_^2^) + (0.1367*P*)^2^ + 0.3086*P*] where *P* = (*F*_o_^2^ + 2*F*_c_^2^)/3
*S* = 1.09	(Δ/σ)_max_ < 0.001
2298 reflections	Δρ_max_ = 0.30 e Å^−^^3^
174 parameters	Δρ_min_ = −0.25 e Å^−^^3^

### Special details

**Table:**

Geometry. All esds (except the esd in the dihedral angle between two l.s. planes) are estimated using the full covariance matrix. The cell esds are taken into account individually in the estimation of esds in distances, angles and torsion angles; correlations between esds in cell parameters are only used when they are defined by crystal symmetry. An approximate (isotropic) treatment of cell esds is used for estimating esds involving l.s. planes.

### Fractional atomic coordinates and isotropic or equivalent isotropic displacement parameters (Å^2^)

**Table:**

	*x*	*y*	*z*	*U*_iso_*/*U*_eq_	
O1	0.2523 (3)	−0.4421 (6)	−0.16266 (9)	0.0723 (9)	
C2	0.1549 (5)	−0.6402 (10)	−0.18009 (16)	0.0753 (13)	
C3	0.1426 (5)	−0.6924 (10)	−0.22703 (16)	0.0762 (13)	
H3	0.082501	−0.819559	−0.246999	0.091*	
C4	0.2352 (5)	−0.5237 (9)	−0.24105 (13)	0.0707 (12)	
H4	0.248630	−0.517334	−0.272006	0.085*	
C5	0.3028 (5)	−0.3696 (9)	−0.20109 (13)	0.0657 (12)	
C6	0.0879 (6)	−0.7575 (13)	−0.1441 (2)	0.1026 (19)	
H6A	0.020336	−0.892412	−0.160150	0.154*	
H6B	0.052540	−0.590266	−0.130271	0.154*	
H6C	0.147806	−0.865048	−0.118645	0.154*	
C7	0.4044 (4)	−0.1635 (8)	−0.19346 (13)	0.0640 (11)	
H7	0.433954	−0.117117	−0.220490	0.077*	
C8	0.4640 (4)	−0.0255 (8)	−0.15184 (12)	0.0650 (11)	
H8	0.438851	−0.064204	−0.123590	0.078*	
C9	0.5671 (4)	0.1829 (8)	−0.15071 (12)	0.0614 (11)	
O10	0.5944 (3)	0.2504 (7)	−0.18937 (9)	0.0766 (10)	
C11	0.6450 (4)	0.3207 (8)	−0.10472 (12)	0.0612 (11)	
C12	0.6189 (5)	0.2587 (9)	−0.05990 (13)	0.0688 (12)	
H12	0.550905	0.132823	−0.058535	0.083*	
C13	0.6944 (5)	0.3854 (11)	−0.01815 (13)	0.0777 (14)	
C14	0.7946 (5)	0.5709 (10)	−0.01895 (13)	0.0773 (14)	
H14	0.845172	0.653156	0.009816	0.093*	
C15	0.8201 (5)	0.6350 (9)	−0.06256 (14)	0.0683 (12)	
C16	0.7460 (4)	0.5130 (9)	−0.10592 (12)	0.0628 (11)	
O17	0.7747 (3)	0.5862 (7)	−0.14744 (9)	0.0786 (9)	
H17	0.724256	0.502456	−0.170370	0.118*	
Cl18	0.94733 (14)	0.8606 (3)	−0.06462 (4)	0.0898 (6)	
Cl19	0.66403 (17)	0.3052 (4)	0.03742 (4)	0.1139 (7)	

### Atomic displacement parameters (Å^2^)

**Table:**

	*U*^11^	*U*^22^	*U*^33^	*U*^12^	*U*^13^	*U*^23^
O1	0.081 (3)	0.0853 (18)	0.0574 (15)	−0.0008 (16)	0.0296 (13)	−0.0014 (12)
C2	0.073 (4)	0.080 (3)	0.076 (3)	−0.001 (2)	0.025 (2)	0.000 (2)
C3	0.072 (4)	0.080 (3)	0.073 (3)	−0.002 (2)	0.012 (2)	−0.0048 (19)
C4	0.075 (4)	0.079 (2)	0.057 (2)	0.000 (2)	0.0147 (18)	−0.0015 (17)
C5	0.072 (4)	0.074 (2)	0.053 (2)	0.012 (2)	0.0199 (17)	0.0040 (15)
C6	0.101 (6)	0.114 (4)	0.108 (4)	−0.012 (3)	0.055 (3)	0.003 (3)
C7	0.066 (4)	0.073 (2)	0.054 (2)	0.005 (2)	0.0184 (17)	0.0058 (15)
C8	0.077 (4)	0.071 (2)	0.0495 (19)	0.002 (2)	0.0218 (17)	0.0082 (15)
C9	0.070 (4)	0.069 (2)	0.0466 (19)	0.0047 (19)	0.0188 (17)	0.0031 (14)
O10	0.090 (3)	0.0963 (19)	0.0489 (15)	−0.0065 (16)	0.0272 (13)	0.0015 (12)
C11	0.062 (3)	0.076 (2)	0.0471 (19)	0.0067 (19)	0.0168 (16)	0.0021 (15)
C12	0.074 (4)	0.085 (2)	0.049 (2)	−0.005 (2)	0.0183 (17)	0.0051 (17)
C13	0.092 (4)	0.095 (3)	0.050 (2)	−0.001 (3)	0.025 (2)	0.0022 (19)
C14	0.088 (4)	0.089 (3)	0.052 (2)	−0.001 (3)	0.0136 (19)	−0.0048 (18)
C15	0.071 (4)	0.073 (2)	0.062 (2)	−0.002 (2)	0.0202 (19)	−0.0013 (17)
C16	0.061 (3)	0.076 (2)	0.0537 (19)	0.004 (2)	0.0202 (16)	0.0046 (16)
O17	0.086 (3)	0.0982 (19)	0.0562 (15)	−0.0112 (17)	0.0270 (13)	0.0045 (13)
Cl18	0.0900 (14)	0.0995 (9)	0.0818 (8)	−0.0179 (7)	0.0260 (6)	−0.0063 (5)
Cl19	0.1348 (17)	0.1627 (14)	0.0488 (7)	−0.0341 (10)	0.0326 (7)	−0.0011 (6)

### Geometric parameters (Å, º)

**Table:**

O1—C2	1.358 (6)	C8—H8	0.9300
O1—C5	1.382 (5)	C9—O10	1.248 (4)
C2—C3	1.327 (6)	C9—C11	1.487 (5)
C2—C6	1.495 (6)	C11—C16	1.390 (6)
C3—C4	1.389 (7)	C11—C12	1.404 (5)
C3—H3	0.9300	C12—C13	1.372 (6)
C4—C5	1.361 (5)	C12—H12	0.9300
C4—H4	0.9300	C13—C14	1.363 (6)
C5—C7	1.399 (6)	C13—Cl19	1.734 (4)
C6—H6A	0.9600	C14—C15	1.370 (5)
C6—H6B	0.9600	C14—H14	0.9300
C6—H6C	0.9600	C15—C16	1.391 (5)
C7—C8	1.337 (5)	C15—Cl18	1.712 (5)
C7—H7	0.9300	C16—O17	1.338 (4)
C8—C9	1.439 (6)	O17—H17	0.8200
			
C2—O1—C5	106.9 (3)	C9—C8—H8	120.0
C3—C2—O1	110.0 (4)	O10—C9—C8	119.6 (3)
C3—C2—C6	133.8 (5)	O10—C9—C11	117.9 (4)
O1—C2—C6	116.2 (4)	C8—C9—C11	122.5 (3)
C2—C3—C4	107.9 (4)	C16—C11—C12	119.3 (3)
C2—C3—H3	126.0	C16—C11—C9	119.7 (3)
C4—C3—H3	126.0	C12—C11—C9	121.1 (4)
C5—C4—C3	107.3 (4)	C13—C12—C11	119.4 (4)
C5—C4—H4	126.4	C13—C12—H12	120.3
C3—C4—H4	126.4	C11—C12—H12	120.3
C4—C5—O1	108.0 (4)	C14—C13—C12	121.7 (4)
C4—C5—C7	133.0 (4)	C14—C13—Cl19	118.7 (3)
O1—C5—C7	119.1 (3)	C12—C13—Cl19	119.6 (4)
C2—C6—H6A	109.5	C13—C14—C15	119.3 (4)
C2—C6—H6B	109.5	C13—C14—H14	120.3
H6A—C6—H6B	109.5	C15—C14—H14	120.3
C2—C6—H6C	109.5	C14—C15—C16	121.1 (4)
H6A—C6—H6C	109.5	C14—C15—Cl18	120.4 (3)
H6B—C6—H6C	109.5	C16—C15—Cl18	118.4 (3)
C8—C7—C5	127.5 (4)	O17—C16—C11	122.4 (3)
C8—C7—H7	116.2	O17—C16—C15	118.4 (4)
C5—C7—H7	116.2	C11—C16—C15	119.2 (3)
C7—C8—C9	120.0 (3)	C16—O17—H17	109.5
C7—C8—H8	120.0		
			
C5—O1—C2—C3	−0.2 (5)	C8—C9—C11—C12	−1.7 (6)
C5—O1—C2—C6	178.3 (4)	C16—C11—C12—C13	−0.9 (6)
O1—C2—C3—C4	0.1 (6)	C9—C11—C12—C13	178.7 (4)
C6—C2—C3—C4	−178.0 (6)	C11—C12—C13—C14	0.1 (7)
C2—C3—C4—C5	−0.1 (5)	C11—C12—C13—Cl19	−179.1 (3)
C3—C4—C5—O1	0.0 (5)	C12—C13—C14—C15	0.6 (7)
C3—C4—C5—C7	−178.7 (5)	Cl19—C13—C14—C15	179.7 (3)
C2—O1—C5—C4	0.1 (5)	C13—C14—C15—C16	−0.3 (7)
C2—O1—C5—C7	179.0 (4)	C13—C14—C15—Cl18	−178.6 (4)
C4—C5—C7—C8	−179.8 (4)	C12—C11—C16—O17	−178.7 (4)
O1—C5—C7—C8	1.6 (7)	C9—C11—C16—O17	1.6 (6)
C5—C7—C8—C9	−179.6 (4)	C12—C11—C16—C15	1.2 (6)
C7—C8—C9—O10	5.1 (6)	C9—C11—C16—C15	−178.5 (4)
C7—C8—C9—C11	−174.1 (4)	C14—C15—C16—O17	179.4 (4)
O10—C9—C11—C16	−1.2 (6)	Cl18—C15—C16—O17	−2.3 (6)
C8—C9—C11—C16	178.0 (4)	C14—C15—C16—C11	−0.6 (7)
O10—C9—C11—C12	179.1 (4)	Cl18—C15—C16—C11	177.8 (3)

### Hydrogen-bond geometry (Å, º)

**Table:**

*D*—H···*A*	*D*—H	H···*A*	*D*···*A*	*D*—H···*A*
O17—H17···O10	0.82	1.76	2.489 (4)	147
C4—H4···O10^i^	0.93	2.54	3.272 (6)	135
C7—H7···O10^i^	0.93	2.57	3.359 (4)	143

Symmetry code: (i) −*x*+1, *y*−1/2, −*z*−1/2.
